# Trends of HIV/AIDS knowledge and attitudes among Nigerian women between 2007 and 2017 using Multiple Indicator Cluster Survey data

**DOI:** 10.1186/s12889-022-12865-y

**Published:** 2022-03-04

**Authors:** Enyinnaya Ukaegbu, Raushan Alibekova, Syed Ali, Byron Crape, Alpamys Issanov

**Affiliations:** 1 Department of Medicine, School of Medicine, Nazarbayev University, Kerey and Zhanibek Khans St 5/1, Nur-Sultan, Kazakhstan 010000; 2 Department of Biomedical Sciences, School of Medicine, Nazarbayev University, Kerey and Zhanibek Khans St 5/1, Nur-Sultan, Kazakhstan 010000

**Keywords:** HIV/AIDS, Knowledge, Attitude, Temporal trends, Nigeria

## Abstract

**Background:**

Globally, Nigeria ranks third among the countries with the highest number of People Living with HIV (PLHIV). Given that HIV/AIDS knowledge is a key factor that determines the risk of transmission and certain attitudes towards PLHIV, there is a need to understand the trend of HIV knowledge within the population for the purpose of assessing the progress and outcome of HIV prevention strategies. The aim of the study was to understand the trends of HIV/AIDS knowledge and attitude towards PLHIV between 2007 to 2017 among Nigerian women, and to investigate change in the factors associated with HIV/AIDS knowledge and attitude towards PLHIV over years.

**Methods:**

Data were derived from three Nigerian Multiple Indicator Cluster Surveys (2007, 2011 and 2016–2017) among women aged 15–49 years old from each geo-political zone (South South, South East, South West, North East, North West, North Central) in Nigeria. Participants who did not answer questions related to HIV/AIDS knowledge and attitude were excluded from the study. The final sample sizes were 17,733 for 2007, 26,532 for 2011 and 23,530 for 2017. In descriptive statistics, frequencies represented the study sample, while percentages represented weighted estimates for the population parameters. Rao-Scott chi-square test for complex survey design studies was used to assess bivariable associations. Factors associated with outcome variables were examined using the survey-weighted multivariable logistic regression models for the complex survey design while controlling for potential confounding variables.

**Results:**

There was a relatively high level of HIV/AIDS knowledge level in 2007 and 2016–2017 surveys (64.6 and 64.1%, respectively), however a decrease in HIV/AIDS knowledge trend was observed in 2011 (45.6%). The positive attitude towards PLHIV progressively increased across the years (from 40.5 to 47.0% to 53.5%). Multivariable analysis revealed that women who had a higher educational level, higher wealth index, and lived in urban areas had higher odds for HIV/AIDS knowledge and positive attitude towards PLHIV across the years. In addition, the Northern zones had predominantly higher knowledge and attitude levels.

**Conclusions:**

Our study found increasing tendency for high HIV/AIDS knowledge and positive attitude towards PLHIV over the years. Women’s age, wealth index, education level and residence were consistently associated with knowledge and attitude over the years. There is a need for more pragmatic HIV/AIDS-related knowledge action plan to target to cover all age groups, all geo-political zones while paying close attention to the rural areas and the less educated women. In addition, more replicative studies of HIV/AIDS knowledge and attitude trends is crucial in monitoring of the progress of HIV interventions in the country in the coming years.

## Background

HIV/AIDS remains a major public health challenge globally [1]. In 2019, there were estimated 38 million people living with HIV (PLHIV) worldwide [2, 3]. It is the leading cause of morbidity and mortality in Sub-Saharan African (SSA), accounting for 71% of the global population of PLHIV [4, 5]. The number of women living with HIV are more than men in SSA region, where women account for 58% of population of PLHIV in the region [6].

In Nigeria, the history of HIV/AIDS dates back to 1985 when the first case was diagnosed and reported in Lagos, Nigeria [7]. Nigeria, the most populous country in Africa [8], has 1.9 million PLHIV (with prevalence of 1.4%) between the ages of 15–49 years, making Nigeria the third highest in HIV burden [9, 10]. Lack of knowledge of HIV and its transmission have shown to be a major contributing factor for its spread [11, 12]. There are gender differences in HIV knowledge, as men appear to be more knowledgeable than women [13]. Other factors related to HIV/AIDS knowledge are education and socio-economic status [14]. The level of knowledge is an important predictor for certain unpleasant attributes towards PLHIV. Previous studies have shown that poor knowledge HIV/AIDS is associated with social stigma and negative attitude towards PLHIV [15, 16]. Stigma and discrimination towards PLHIV to a large extent hampers prevention, treatment, and care [17, 18]. Studies conducted in USA, India and Nigeria showed that negative attitude is often due to poor knowledge of HIV in the general population [19–21].

SSA has witnessed some degree of increase in HIV/AIDS knowledge and accepting attitude towards PLHIV since 1990 [22]. HIV knowledge, awareness and testing has increased in recent years in the general Nigerian population probably due to combined efforts of the government and corporate bodies towards HIV prevention [23] in response to the national goal of HIV control captured in the National HIV/AIDS Strategic Plan (2017–2021) [24]. These efforts are linked to the target of 95–95-95 strategy set by the Joint United Nations Programme on HIV/AIDS (UNAIDS) to end HIV epidemic by 2030 [25] while ensuring healthy lives and promoting well-being for all ages – a major focus of the Sustainable Development Goals (SDGs) [26]. Also, a trend study conducted in 2018 demonstrated seemingly increasing HIV-related knowledge [27]. However, there is dearth of studies on trend in HIV/AIDS- related knowledge among women and their attitude towards PLHIV in Nigeria. The outcome of this study will be helpful in assessing the progress made over the years to improve knowledge regarding HIV/AIDS among reproductive aged Nigerian female population. The aim of this study was to assess the temporal trends of HIV/AIDS knowledge and attitude towards PLHIV between 2007 and 2017 and to investigate change in factors associated with HIV/AIDS knowledge and attitude towards PLHIV over years among Nigerian women using the secondary data from the Multiple Indicator Cluster Surveys (MICS).

## Methods

### Study design

The study utilized secondary data collected through three separate cross-sectional studies [28] conducted between March and April in 2007, between February and March 2011, and between September 2016 and January 2017 in the thirty-six (36) states of the Federation and the Federal Capital Territory in Nigeria. The cross-sectional studies were based on the comprehensive MICS study conducted by the National Bureau of Statistics (NBS) with financial and technical support from United Nations Children’s Fund (UNICEF), World Health Organization (WHO), World Bank, and United Nations among others [29–31]. The global MICS program was developed to provide sound statistical and internationally comparable data on several indicators of health situation of women and children (including knowledge of HIV and AIDS, reproductive health literacy and education among others). The MICS data allows countries to generate convincing evidence for policy use and to monitor progress towards national goals and targets arising from the internationally agreed-upon commitments.

### Sampling method and study population

A two-stage stratified cluster sampling technique was applied to select participants for each study. This involved mapping the states within each geo-political zone (South South, South East, South West, North East, North West, North Central) as main sampling strata. Using the National population census, states were divided into Enumeration Areas (EAs). The Enumeration Areas (between 30 and 60) selected within each state were the primary sampling units, and within each EA households were sampled both using systematic sampling approach.

**Fig. 1:**
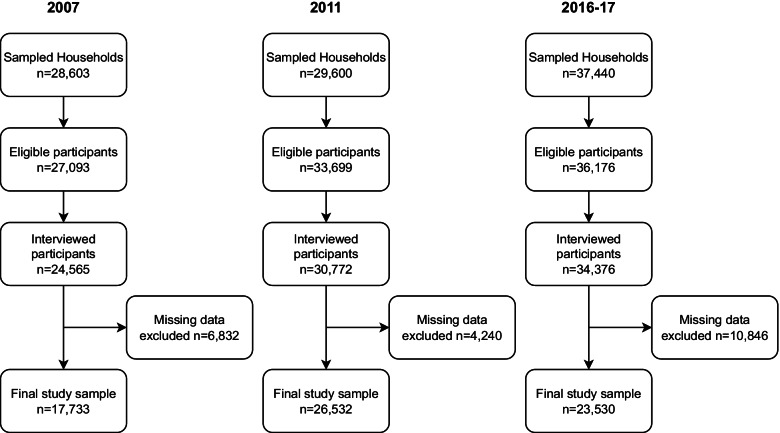
Flow chart representing the inclusion and exclusion process of participants from the Multiple Indicator Cluster Surveys 2007, 2011, and 2016–2017 among women 15–49 years in Nigeria

The study population included only women between 15 and 49 years of age. All the participants who ‘have heard of HIV/AIDS’ were part of the study and the question served as the inclusion criteria for the study. For our study, male respondents, children (under 5 years) were excluded. Also, respondents who did not answer questions related to HIV/AIDS knowledge and attitude were excluded from our study sample (Fig. 1). The MICS study in 2007 which was the third in the series of MICS study in Nigeria had 85.3% response rate. The 2011 survey had a 91% response rate while 2016–2017 survey had a response rate of 95.0%. The final sample sizes were 17,733 for MICS 2007, 26,532 for MICS 2011 and 23,530 for MICS 2016–2017.

### Data collection

The survey data were obtained through face-to-face interviews. The survey data included socio-demographic, sexual behavior, knowledge, attitude, and practice about HIV transmission characteristics. The questionnaire was adapted based on the generic English version of MICS three, four and five model questionnaires. The questionnaire was pre-tested in different states of the Federation with field staff who were fluent in local languages, and familiar with culture and beliefs of the inhabitants of the communities in the selected states. After pre-testing, modifications were made for clarity of wording and order of questions in the survey [32].

### Outcome variables

The study outcomes variables were HIV/AIDS knowledge and attitude towards PLHIV. The binary outcome HIV/AIDS knowledge variable was created using the responses to questions on HIV/AIDS knowledge (Table 1). Responses ‘do not know’, ‘not sure’, or ‘it depends on’ were categorized as ‘no’ (meaning that a participant probably did not know what was the correct answer). The correct response was coded one (1) and incorrect as zero (0). The maximum scores for knowledge were 12 (MICS 2007) and 11 (MICS 2011 and 2016–2017). Based on HIV expert opinions and distribution of HIV/AIDS knowledge scores, HIV/AIDS knowledge was dichotomized as ‘high knowledge’ and ‘low knowledge’ groups. We used the HIV knowledge cut-off level of > 8 for “high knowledge” and ≤ 8 for “low knowledge” for MICS 2007, while for MICS 2011 and 2016–2017, we utilized > 7 for “high knowledge” and ≤ 7 for “low knowledge” as the number of knowledge questions were lower than in MICS 2007.

**Table 1:** Descriptive statistics of HIV/AIDS knowledge among women 15-49 years in Nigeria using the Multiple Indicator Cluster Surveys 2007, 2011, and 2016–2017

Knowledge questions – ‘Yes’ (%)^a^	2007 (***n*** = 17,733)	2011 (***n*** = 26,532)	2017 (***n*** = 23,530)
Can avoid AIDS by having one uninfected partner	81.7	80.5	84.5
Can get AIDS through supernatural means	64.2	68.2	73.1
Can avoid AIDS by using a condom correctly every time	56.8	60.8	71.1
Can get AIDS from mosquito bites	64.9	66.6	72.4
Can get AIDS by sharing food with person with AIDS virus	59.7	75.0	73.2
Healthy-looking person to have AIDS	75.7	68.7	82.2
AIDS from mother to child during pregnancy	92.5	66.9	83.0
AIDS from mother to child at delivery	72.1	68.0	83.6
AIDS from mother to child through breastmilk	78.0	78.7	93.1
AIDS virus prevented from unborn child	–	57.7	76.5
Can avoid AIDS by not having sex at all	71.9	–	–
Can get AIDS by injection with needle already used by someone	80.1	–	–

*Abbreviations*: *HIV* human immunodeficiency virus, *AIDS* acquired immunodeficiency syndrome

^a^Responses to knowledge questions were dichotomous (“Yes” or “No”)

**Fig. 2:**
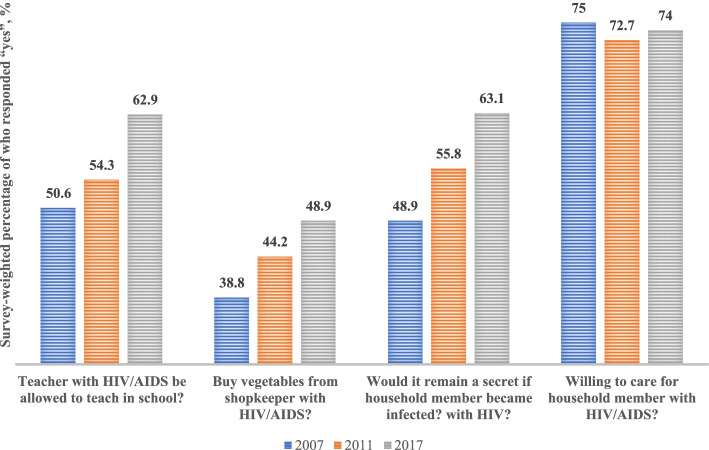
Survey-weighted percentages of respondents who positively responded to questions assessing attitude towards people living with HIV using the Multiple Indicator Cluster Surveys 2007, 2011, and 2016–2017 among women 15–49 years in Nigeria. Positive response is considered when a respondent answered “yes” to these questions

The binary attitude towards PLHIV variable was created with the four attitude questions (Fig. 2). The accepting response was coded as one (1) and dismissing responses as zero (0). Scores from four questions were summed and the attitude towards PLHIV variable was categorized as ‘positive attitude’ (> 2) and ‘negative attitude’ (<=2), again based on the experts’ opinions and the distribution of attitude scores.

### Independent variables

The independent variables (socio-demographic characteristics) included age (15–19, 20–24, 25–29, 30–34, 35–39, 40–44, 45–49); marital status (currently married, formerly married, never married); educational level and wealth index. Educational level was categorized to (none, non-formal (education outside the school system), primary school, secondary (high school) and above (tertiary educational institutions). Wealth index was calculated based on the economic status of the participant measured through household information about ownership of consumer goods and household characteristics, and categorized based on quantiles into [poorest, second (poor), middle, fourth (rich), richest]. Also, geographical variables were collected including area of residence (urban/rural) and the six geo-political (administrative) zones. Based on the available dataset, only independent variables available for the individual categories for the three surveys were used for the analysis to allow for consistency among the three surveys.

### Statistical analysis

The secondary data were obtained from the MICS UNICEF online database [33] and sample weights were used in all the analyses to adjust for disproportionate sampling so as to obtain less biased population parametric estimates. In descriptive statistics, frequencies represented the study sample, while percentages represented weighted estimates for the population parameters. The Rao-Scott chi-square test for complex survey design studies was used to assess bivariable associations [34]. A survey-weighted multiple logistic regression analysis was performed to investigate the relationship between the outcomes and independent variables. Models were built by adding each individual independent variable based on the statistical significance by using the likelihood-ratio test and the Akaike and Bayesian information criteria until a robust predictive model was arrived at. We checked for multi-collinearity (using variance inflation factor) and interaction of the independent variables. We used the Archer-Lemeshow test for design-based regression models to test goodness of fit of the model [35].

To test the internal reliability of the knowledge and attitude outcomes, Cronbach alphas were calculated. The Cronbach alphas for the HIV knowledge were 0.66, 0.67, and 0.54 for 2007, 2011 and 2016–2017 respectively; and Cronbach alphas for attitude towards PLHIV were 0.57, 0.63, and 0.64 for 2007, 2011 and 2016–2017 respectively. Two-sided *p*-value less than 0.05 indicated statistical significance. All the analysis was done analysis using STATA 16 Corp software.

## Results

### Socio-demographic characteristics

Socio-demographic characteristics of the study participants are presented in Table 2. Majority of the participants were ‘currently married’ and from the rural area across the three surveys. Respondents with the secondary education and above were 1.2% in 2007, 55.2% in 2011 and 13.1% in 2017.

**Table 2:** Socio-demographic characteristics of the study respondents of the Multiple Indicator Cluster Surveys 2007, 2011, and 2016–2017 among women 15–49 years in Nigeria

Variables	2007 (***n*** = 17,733), ***n*** (%)^a^	2011 (***n*** = 26,532) ***n*** (%)^a^	2017 (***n*** = 23,530) ***n*** (%)^a^
**Age group, n (%)**			
15–19	3030 (17.4)	4564 (17.6)	4201 (17.9)
20–24	3194 (18.0)	4541 (17.1)	3922 (17.1)
25–29	3530 (20.1)	5002 (19.4)	4150 (17.7)
30–34	2793 (16.0)	3947 (15.9)	3838 (16.5)
35–39	2277 (12.8)	3177 (12.3)	3077 (12.9)
40–44	1699 (8.9)	2631 (10.0)	2545 (10.5)
45–49	1210 (6.7)	2079 (7.8)	1797 (7.4)
**Marital status**^b^			
Currently married	12,191 (67.6)	18,242 (69.0)	16,101 (70.1)
Formerly married	776 (4.4)	1182 (4.7)	1089 (4.5)
Never married	4766 (28.0)	6517 (26.3)	6299 (25.5)
**Educational level**			
None	5875 (29.4)	8247 (26.9)	3758 (18.0)
Non-standard curriculum	3669 (20.9)	–	3673 (14.9)
Primary	7843 (48.6)	4964 (17.9)	10,529 (41.4)
Secondary and above	346 (1.2)	12,730 (55.2)	3249 (13.1)
Higher	–	–	2321 (12.6)
**Wealth index quantile**			
Poorest	2602 (12.3)	5179 (15.0)	2615 (12.2)
Second	3208 (15.4)	5727 (17.3)	3855 (15.9)
Middle	3566 (19.4)	5458 (19.8)	4884 (20.4)
Fourth	4080 (23.9)	5084 (22.6)	5611 (23.5)
Richest	4277 (29.1)	4493 (25.2)	6565 (28.0)
**Area**			
Rural	13,388 (61.8)	19,530 (59.8)	14,917 (58.9)
Urban	5581 (38.2)	7002 (40.2)	8613 (41.1)
**Zone (%)**			
North Central	3650 (13.1)	5356 (14.5)	4984 (17.5)
North East	1857 (7.9)	4535 (12.7)	2837 (17.2)
North West	3819 (22.0)	4783 (19.6)	5368 (28.3)
South East	2687 (12.1)	3471 (13.1)	2842 (8.1)
South South	3269 (18.1)	3824 (16.7)	4091 (13.4)
South West	2451 (26.8)	3972 (23.4)	3408 (15.4)

^a^Frequencies refer to the study sample, while percentages are survey-weighted estimates for the Nigerian female population

^b^Currently married included participants who were currently married or living with their partner. Formerly married included participants who were widowed, divorced, or separated. Never married included participants who were never married or single

We explored the number of participants who have been tested for HIV (‘ever had HIV test?’). Notably, 10.0% of respondents indicated that they have been tested in 2007, in 2011; 23.5% and in 2017; 41.1% of respondents indicated that they have had HIV test.

### HIV/AIDS knowledge

In general, 64.6% of the respondents in 2007 had high HIV/AIDS knowledge level, in 2011 it dropped to 45.6%, and in 2017 increased to 64.1%. The percentage of high HIV/AIDS knowledge increased with the higher wealth index quantiles (‘fourth’ and ‘richest’). Respondents who had higher secondary education, who were in richest wealth index and live-in urban areas had the highest knowledge level across all the three surveys. The Northwest zone had the highest knowledge level (70.7%) in 2007 compared to other zones, however, Southwest had the highest knowledge level among the zones in 2011 and 2017 (Table 3). Although there was an overall decrease in knowledge level in 2011, all the independent variables showed a considerable increase in 2017.

**Table 3:** Socio-demographic characteristics of the study participants stratified by HIV/AIDS knowledge level and attitude towards people living with HIV using the Multiple Indicator Cluster Surveys 2007, 2011, and 2016–2017 among women 15–49 years in Nigeria

Variable	High Knowledge (%)			Positive attitude (%)		
	2007 (64.6%)	2011 (45.6%)	2017 (64.1%)	2007 (40.5%)	2011 (47.0%)	2017 (53.5%)
**Age group**						
15–19	62.2%	41.7%	62.3%	40.4%	48.0%	53.3%
20–24	67.7%	44.9%	64.4%	44.4%	49.0%	56.2%
25–29	66.6%	47.3%	65.1%	41.3%	48.5%	54.4%
30–34	65.0%	48.1%	65.5%	40.7%	47.3%	52.3%
35–39	65.6%	47.5%	65.0%	59.4%	45.0%	52.3%
40–44	58.4%	44.6%	65.4%	35.8%	43.5%	52.4%
45–49	61.7%	44.6%	61.2%	33.6%	44.1%	52.3%
**Marital status**						
Currently married	63.8%	44.6%	62.7%	39.5%	44.7%	52.1%
Formerly married	61.0%	45.8%	69.5%	35.7%	47.6%	57.5%
Never married	67.1%	48.0%	67.0%	43.6%	53.0%	56.8%
**Education**						
None	56.3%	30.5%	53.2%	39.9%	41.5%	51.9%
Primary	60.6%	40.8%	60.8%	32.4%	40.9%	52.2%
Secondary and above	71.3%	54.5%	65.3%	44.1%	51.7%	66.4%
**Wealth index quantile**						
Poorest	52.2%	26.7%	53.2%	40.8%	40.1%	50.5%
Second	57.4%	34.9%	56.6%	38.6%	40.7%	52.2%
Middle	63.6%	45.4%	60.6%	37.7%	45.9%	51.9%
Fourth	64.9%	51.4%	65.3%	36.5%	46.6%	54.3%
Richest	74.0%	59.0%	75.3%	46.5%	56.8%	56.2%
**Area**						
Rural	60.8%	39.8%	58.1%	38.8%	43.3%	52.3%
Urban	70.7%	54.2%	72.6%	43.1%	52.5%	55.3%
**Zone**						
North Central	60.7%	34.1%	59.2%	42.2%	51.8%	54.5%
North East	46.5%	34.6%	65.2%	39.9%	48.7%	62.9%
North West	70.7%	41.7%	63.7%	52.4%	52.4%	59.6%
South East	66.8%	49.1%	62.0%	38.1%	46.4%	46.8%
South South	63.3%	53.0%	65.8%	32.5%	48.1%	54.2%
South West	66.7%	54.6%	68.8%	36.6%	38.3%	33.8%

In the multivariable logistic regression analysis, age was associated with high knowledge across all three surveys. The participants in 35–39 age category had the highest adjusted odds of high knowledge level in 2007 (aOR = 1.45, 95%CI: 1.22 to 1.73, *p* < 0.001); in 2011, age category 45–49 had the highest odds (aOR = 1.50, 95%C. I: 125 to 1.80, *p* < 0.001) and in 2017, 40–44 years old had the highest odds the high knowledge (aOR = 1.28, 95%C. I: 1.06 to 1.55, *p* = 0.01) (Table 4). Participants with secondary and above educational level showed a higher odd for high knowledge level after adjusting for other covariates in 2011 (aOR = 2.05, 95% CI: 1.76 to 2.38, *p* < 0.001); in 2017 (aOR = 2.01, 95% CI: 1.63 to 2.46, *p* < 0.001), respectively. However, this was different for 2007, as primary educational level had the highest odds for high knowledge (aOR = 2.01; 95% CI:1.73 to 2.34, *p* < 0.001). Participants who lived in urban area and those with higher wealth index quantiles were associated with high knowledge. Also, after controlling for other covariates, Northwest zone consistently had higher the odds of high knowledge than any other zone across all years; in 2007 (aOR = 2.22, 95% CI: 1.83 to 2.70, *p* < 0.001), in 2011 (aOR = 2.22, 95% CI: 1.74 to 2.85, *p* < 0.001) and in 2017 (aOR = 1.66, 95% CI: 1.39 to 1.99, *p* = < 0.001). In addition, we observed that the participants who have tested for HIV have statistically significant higher odds for HIV knowledge than those who have not been tested.

**Table 4:** Survey-weighted multivariable logistic regression analysis to determine factors associated with HIV/AIDS knowledge level over years using the Multiple Indicator Cluster Surveys 2007, 2011, and 2016–2017 among women 15–49 years in Nigeria

Variable	2007		2011		2017	
	uOR (95% CI)	aOR^**†**^ (95% CI)	uOR (95% CI)	aOR^**†**^ (95% CI)	uOR^**†**^ (95% CI)	aOR^**†**^ (95% CI)
**Age group**						
15–19	Ref	Ref	Ref	Ref	Ref	Ref
20–24	1.27 (1.11;1.45)**	1.34 (1.16;1.55)***	1.14 (1.01;1.28)*	1.25 (1.12;1.40)***	1.10 (0.97;1.24)	1.03 (0.89;1.19)
25–29	1.21 (1.06;1.39)**	1.34 (1.15;1.57)***	1.26 (1.11;1.43)***	1.32 (1.14;1.52)***	1.13 (1.00;1.28)*	1.07 (0.91;1.26)
30–34	1.13 (0.98;1.29)	1.31 (1.10;1.56)**	1.29 (1.15;1.46)***	1.43 (1.22;1.67)***	1.10 (0.97;1.24)	0.99 (0.84;1.17)
35–39	1.16 (1.01;1.34)*	1.45 (1.22;1.73)***	1.26 (1.12;1.43)***	1.37 (1.17;1.60)***	1.13 (0.99;1.28)	1.11 (0.93;1.33)
40–44	0.85 (0.74;0.99)*	1.15 (0.95;1.38)	1.13 (0.98;1.29)	1.41 (1.19;1.68)***	1.15 (1.00;1.32)	1.28 (1.06;1.55)*
45–49	0.98 (0.84;1.15)	1.42 (1.15;1.76)**	1.12 (0.97;1.30)	1.50 (1.25;1.80)***	0.96 (0.82;1.11)	1.08 (0.89;1.31)
**Marital status**						
Currently married	Ref	Ref	Ref	Ref	Ref	Ref
Formerly married	0.89 (0.75;1.06)	0.99 (0.82;1.19)	1.05 (0.89;1.23)	0.94 (0.78;1.13)	1.35 (1.15;1.60)***	1.30 (1.08;1.57)***
Never married	1.16 (1.05;1.28)**	1.13 (0.97;1.30)	1.14 (1.04;1.26)**	1.08 (0.93;1.25)	1.21 (1.10;1.33)***	1.43 (1.25;1.64)***
Educational level*						
None	Ref	Ref	Ref	Ref	Ref	Ref
Non-formal	1.19 (1.06;1.34)**	1.39 (1.21;1.60)***	–	–	1.19 (1.04;1.37)*	1.08 (0.92;1.26)
Primary	1.92 (1.72;2.15)***	2.01 (1.73;2.34)***	1.57 (1.37;1.79)***	1.37 (1.18;1.58)***	1.61 (1.40;1.85)***	1.24 (1.06;1.45)**
Secondary and above	1.56 (1.06;2.30)*	1.22 (0.83;1.80)	2.73 (2.39;3.11)***	2.05 (1.76;2.38)***	3.68 (3.09;4.38)***	2.01 (1.64;2.46)***
Higher	–	–	–	–	1.13 (0.94;1.36)	1.06 (0.87;1.31)
**Wealth Index**						
Poorest	Ref	Ref	Ref	Ref	Ref	Ref
Second	1.23 (1.05;1.45)*	1.12 (0.96;1.32)	1.47 (1.27;1.70)***	1.32 (1.11;1.59)**	1.10 (0.95;1.29)	1.02 (0.88;1.19)
Middle	1.59 (1.33;1.91)***	1.41 (1.18;1.68)***	2.29 (1.98;2.64)***	1.71 (1.40;2.08)***	1.35 (1.13;1.62)**	1.07 (0.88;1.30)
Fourth	1.69 (1.41;2.02)***	1.43 (1.18;1.74)***	2.90 (2.48;3.39)***	1.74 (1.40;2.18)***	1.65 (1.40;1.95)***	1.03 (0.84;1.26)
Richest	2.61 (2.16;3.15)***	1.95 (1.56;2.43)***	3.94 (3.38;4.59)***	1.99 (1.60;2.46)***	2.68 (2.28;3.15)***	1.31 (1.06;1.63)*
**Area**						
Rural	Ref	Ref	Ref	Ref	Ref	Ref
Urban	1.56 (1.37;1.77)***	1.09 (0.93;1.27)	1.79 (1.57;2.03)***	1.20 (0.99;1.45)	1.91 (1.70;2.15)***	1.40 (1.21;1.61)***
**Zone**						
North Central	Ref	Ref	Ref	Ref	Ref	Ref
North East	0.56 (0.44–0.73)***	0.82 (0.64;1.04)	1.02 (0.75;1.40)	1.79 (1.34;2.40)***	1.29 (1.04;1.60)*	1.61 (1.32;1.98)***
North West	1.57 (1.29;1.90)***	2.22 (1.83;2.70)***	1.38 (1.11;1.72)**	2.22 (1.74;2.85)***	1.21 (1.03;1.42)*	1.66 (1.39;1.99)***
South East	1.30 (1.08;1.58)**	0.90 (0.74;1.08)	1.86 (1.58;2.19)***	1.61 (1.36;1.90)***	1.12 (0.96;1.31)	0.92 (0.78;1.07)
South South	1.12 (0.92;1.36)	0.84 (0.69;1.02)	2.18 (1.82;2.60)***	1.79 (1.50;2.15)***	1.32 (1.13;1.55)***	1.14 (0.97;1.35)
South West	1.30 (1.07;1.57)**	0.84 (0.70;1.02)	2.32 (1.98;2.73)***	1.61 (1.34;1.93)***	1.52 (1.29;1.79)***	1.07 (0.90;1.27)
**HIV Testing**						
No	Ref	Ref	Ref	Ref	Ref	Ref
Yes	1.55 (1.34;1.81)***	1.27 (1.08;1.49)**	2.10 (1.89;2.32)***	1.50 (1.36;1.66)***	2.30 (2.09;2.52)***	2.16 (1.93;2.41)***

*Abbreviations*: *uOR* unadjusted odds ratio, *aOR* adjusted odds ratio, *CI* confidence interval, *Ref* Reference group, *HIV* human immunodeficiency virus

^**†**^*P*-values of the Archer Lemeshow test statistics for design-based regression models for the ‘HIV knowledge’ outcome variable were 0.04 for 2007, 0.02 for 2011 and 0.05 for 2017

*p < 0.05, **p < 0.01, ***p < 0.001

### Attitude towards PLHIV

Overall, proportions of respondents with positive attitude towards PLHIV progressively increased from 40.5% in 2007 to 47.0% in 2011 and to 53.5% in 2017. However, respondents who were in ‘poorest’ wealth index quantile showed a decline in high knowledge in 2011 with an increase in 2017 (Table 3). The South South zone had the least percentage of attitude level in 2007 (32.5%), and Southwest the least in 2011 (38.3%) and in 2017 (33.8%). An interesting finding after analyzing the individual attitude questions indicates that majority of the respondents ‘who would buy vegetables from shopkeeper with HIV/AIDS’ was consistently lower across the years, as compared to those ‘who are willing to care for a person with HIV/AIDS in a household’ (Fig. 2).

In multivariable analysis of attitude, age was associated with positive attitude across all three surveys. Participants who were ‘never married’ had higher odds for positive attitude than currently married in 2007 (aOR = 1.34, 95% CI: 1.18 to 1.56, *p* < 0.001), in 2011 (aOR = 1.64, 95% CI: 1.42 to 1.90, *p* < 0.001) and in 2017 (aOR = 1.62, 95% CI: 1.42 to 1.85, *p* < 0.001). Participants with secondary and above educational level showed a higher odd for high knowledge level after adjusting for other covariates in 2011 and in 2017. However, this was different for 2007 with primary educational level having the highest odds for high knowledge (aOR = 1.81; 95% CI: 1.54 to 2.12, *p* < 0.001). After adjusting for other covariates, participants in wealth index ‘richest’ and urban area were associated with positive attitude towards PLHIV. All the Northern zones had higher odds of positive attitude across all surveys (Table 5). In addition, we observed that the participants who have tested for HIV have statistically significant higher odds for attitude towards PLHIV than respondents who have not been tested.

**Table 5:** Survey-weighted multivariable logistic regression analysis to determine factors associated with attitude towards people living with HIV over years using the Multiple Indicator Cluster Surveys 2007, 2011, and 2016–2017 among women 15–49 years in Nigeria

Variable	2007		2010		2017	
	uOR (95% CI)	aOR^**†**^ (95% CI)	uOR (95% CI)	aOR^**†**^ (95% CI)	uOR (95% CI)	aOR^**†**^ (95% CI)
**Age group**						
15–19	Ref	Ref	Ref	Ref	Ref	Ref
20–24	1.18 (1.04;1.34)**	1.23 (1.08;1.40)**	1.04 (0.93;1.16)	1.19 (1.05;1.35)**	1.12 (1.00;1.27)	1.06 (0.93;1.21)
25–29	1.04 (0.92;1.18)	1.21 (1.05;1.40)**	1.02 (0.91;1.14)	1.26 (1.09;1.46)**	1.04 (0.93;1.17)	0.97 (0.83;1.13)
30–34	1.01 (0.88;1.16)	1.21 (1.03;1.42)*	0.97 (0.84;1.12)	1.31 (1.11;1.55)**	0.96 (0.86;1.07)	1.02 (0.87;1.20)
35–39	1.01 (0.88;1.16)	1.32 (1.11;1.56)**	0.89 (0.78;1.01)	1.22 (1.03;1.44)*	0.96 (0.84;1.10)	1.08 (0.90;1.29)
40–44	0.82 (0.71;0.96)*	1.09 (0.91;1.31)	0.83 (0.73;0.95)**	1.27 (1.07;1.50)**	0.96 (0.85;1.09)	1.18 (0.99;1.40)
45–49	0.75 (0.63;0.89)***	1.10 (0.90;1.33)	0.86 (0.73;1.00)	1.33 (1.12;1.59)**	0.96 (0.82;1.12)	1.24 (1.02;1.51)*
**Marital status**						
Currently married	Ref	Ref	Ref	Ref	Ref	Ref
Formerly married	0.85 (0.71;1.02)	1.14 (0.95;1.36)	1.12 (0.94;1.34)	1.26 (1.03;1.55)*	1.24 (1.06;1.45)**	1.29 (1.06;1.58)*
Never married	1.18 (1.06;1.32)**	1.35 (1.18;1.56)***	1.40 (1.27;1.53)***	1.64 (1.42;1.90)***	1.20 (1.11;1.31)***	1.62 (1.42;1.85)***
**Educational level**						
None	Ref	Ref	Ref	Ref	Ref	Ref
Non-formal	0.75 (0.64;0.86)***	1.24 (1.05;1.46)**	–	–	0.90 (0.78;1.03)	1.07 (0.92;1.24)
Primary	1.19 (1.03;1.36)*	1.81 (1.54;2.12)***	0.98 (0.86;1.11)	1.21 (1.05;1.39)*	1.01 (0.88;1.16)	1.25 (1.06;1.46)**
Secondary and above	0.97 (0.69;1.38)	0.80 (0.55;1.17)	1.51 (1.35;1.70)***	1.50 (1.29;1.74)***	1.83 (1.55;2.16)***	2.19 (1.80;2.67)***
Higher	–	–	–	–	1.01 (0.86;1.19)	0.94 (0.78;1.13)
**Wealth Index**						
Poorest	Ref	Ref	Ref	Ref	Ref	Ref
Second	0.91 (0.76;1.09)	0.94 (0.78;1.14)	1.02 (0.90;1.17)	1.02 (0.89;1.19)	1.07 (0.91;1.26)	1.05 (0.89;1.24)
Middle	0.88 (0.71;1.08)	0.95 (0.75;1.19)	1.26 (1.07;1.49)**	1.27 (1.06;1.53)**	1.06 (0.89;1.25)	0.96 (0.79;1.16)
Fourth	0.83 (0.69;1.01)	1.01 (0.80;1.27)	1.30 (1.10;1.53)**	1.39 (1.14;1.70)**	1.17 (0.98;1.39)	1.04 (0.82;1.33)
Richest	1.26 (1.03;1.54)*	1.49 (1.16;1.92)**	1.96 (1.67;2.31)***	1.95 (1.59;2.39)***	1.26 (1.06;1.49)**	1.22 (0.96;1.54)
**Area**						
Rural	Ref	Ref	Ref	Ref	Ref	Ref
Urban	1.20 (1.04;1.37)*	1.00 (0.84;1.18)	1.44 (1.25;1.66)***	1.38 (1.18;1.62)***	1.13 (1.01;1.26)*	1.14 (0.97;1.34)
**Zone**						
North Central	Ref	Ref	Ref	Ref	Ref	Ref
North East	0.91 (0.71;1.16)	1.24 (0.97;1.59)	0.88 (0.72;1.09)	1.39 (1.13;1.71)**	1.41 (1.18;1.70)***	1.83 (1.45;2.30)***
North West	1.51 (1.23;1.85)***	2.08 (1.70;2.54)***	1.03 (0.84;1.25)	1.57 (1.29;1.90)***	1.23 (1.06;1.43)**	1.73 (1.47;2.04)***
South East	0.84 (0.69;1.02)	0.58 (0.47;0.71)***	0.81 (0.67;0.97)*	0.67 (0.57;0.80)***	0.73 (0.62;0.87)***	0.56 (0.47;0.67)***
South South	0.66 (0.55;0.79)***	0.50 (0.41;0.60)***	0.86 (0.70;1.06)	0.65 (0.53;0.80)***	0.99 (0.85;1.15)	0.79 (0.67;0.93)**
South West	0.79 (0.65;0.96)*	0.56 (0.46;0.69)***	0.58 (0.47;0.71)***	0.34 (0.28;0.41)***	0.43 (0.37;0.49)***	0.30 (0.25;0.35)***
**HIV Testing**						
No	Ref	Ref	Ref	Ref	Ref	Ref
Yes	1.63 (1.43;1.87)***	1.66 (1.44;1.90)***	1.80 (1.62;2.00)***	1.62 (1.45;1.81)***	1.93 (1.75;2.14)***	2.21 (1.99;2.46)***

*Abbreviations*: *uOR* unadjusted odds ratio, *aOR* adjusted odds ratio, *CI* confidence interval, *Ref* Reference group, *HIV* human immunodeficiency virus

^**†**^*P*-values of the Archer Lemeshow test statistics for design-based regression models for the ‘attitude towards PLHIV’ outcome variable were 0.32 for 2007, 0.09 for 2011 and 0.22 for 2017

*p < 0.05, **p < 0.01, ***p < 0.001

## Discussion

In this study, we aimed to assess the trends of HIV knowledge and attitude towards PLHIV among Nigerian women. To the best of our knowledge, this is the first study to assess the trends of HIV knowledge and attitude towards PLHIV among Nigerian women using the MICS study. We observed a relatively high level of HIV/AIDS knowledge level in 2007 (64.6%) and 2016–2017 (64.1%). This relatively high knowledge level observed in our study gives credence to studies that examined trends of HIV-related knowledge awareness within SSA region [22, 36]. Our study did not focus on the reason behind the changing levels of HIV/AIDS knowledge. However, the decline in 2011 survey by 19% from the 2007 survey could be attributable to inadequacies in HIV/AIDS awareness and implementation plan. The upwards trajectory in knowledge level seen in 2016–2017 survey might be due to the country’s intervention strategies, which include the introduction of the HIV/AIDS National Strategic Plan (NSP) 2010–2015 and the National HIV/AIDS Stigma Reduction Strategy 2016 [23, 37]. These country efforts might have also reflected the progressive increase in attitude towards PLHIV observed over the years in our study.

An interesting finding in this study revealed that the percentages of people ‘who know that correct usage of condoms would protect from contracting HIV’ increased over the years, in addition to ‘those who knew that HIV could not be transmitted through supernatural means’ (Table 1). This could imply that there is increasing awareness of HIV/AIDS, because the knowledge of condom use is an important tool in the strategy towards HIV prevention [38]. Despite the increasing high HIV/AIDS knowledge level revealed in our study, there could be the existence of increased risky behaviors among population, as high knowledge of HIV/AIDS does not equate to the adoption of preventive sexual behaviors [39].

In the multivariable analysis, age was associated with high HIV knowledge level. Women greater than 40 years statistically significant showed a higher likelihood for higher knowledge. A study in Vietnam found that participants less than 40 years had higher odds for high knowledge than older women, suggesting that younger people may be more interested in HIV/AIDS information because they are in their sexually active years [40]. However, this assumption was statistically validated in our 2007 survey results. Some associations were seen between educational level and area of residence with high HIV/AIDS knowledge level across the three surveys. Women with secondary and above educational level and those who lived in ‘urban’ areas were more likely to have higher HIV/AIDS knowledge. These findings are consistent with a previous study [40].

Furthermore, in this study wealth index was a strong predictor of HIV knowledge and attitude towards PLHIV among women. This corroborated the results reported in a previous study in Nigeria [41, 42] where the richest respondents had higher odds for HIV knowledge and attitude towards PLHIV than those in the poorest category. After adjusting for covariates, Northwest zone showed consistently higher odds for HIV/AIDS knowledge level amongst other zones across all years, indicative of better organizational HIV/AIDS education and awareness activities than other zones. The differences in HIV awareness in the geo-political zones may be due to political dynamics that play a role in multi-ethnic countries such as Nigeria, thus raising a major concern regarding the equity in the redistribution of administrative resources [43, 44]. HIV interventions should be modified to ensure that all zones are equally accessed notwithstanding the socio-political peculiarity of the zone.

Overall, the study shows an increasing rate in positive attitude towards PLHIV over the years. This progressive trend in positive attitude towards PLHIV did not correspond to our finding on HIV/AIDS knowledge trend as seen with the knowledge drop in 2011. This might be because of effects of social desirability responses of which respondents answered questions based on the socially acceptable responses [45]. Furthermore, some associations were seen in attitude towards PLHIV. Respondents with higher educational level, those residing in urban areas and those with a higher wealth index had a more positive attitude towards PLHIV. This finding lends credence to a previous study [42]. There is need for improved HIV/AIDS educational programs and resources targeted at people with educational level below secondary school and in areas of high wealth inequalities. Majority of the population were willing to take care of a household member with HIV (Fig. 2), indicating some level of empathy for HIV positive family members. In addition, most participants in our study expressed aversion towards food/vegetables vendors with HIV (Fig. 2) which may suggest an existence of some form of negative attitudes towards PLHIV, both consistent with a previous study in Nigeria [41].

For effective diagnosis and care of HIV population, there is a growing need for testing [46]. Notably, our study showed a progressively increasing HIV testing across the surveys and was associated with high knowledge level and positive attitude towards PLHIV. A study in South Africa revealed that HIV/AIDS knowledge and having tested for HIV is associated with reduction of stigma [47]. Stigma has been identified as a barrier for effective strategies in the planning and implementation of HIV/AIDS prevention program [48, 49]. Pragmatic efforts should therefore be made at eliminating these barriers that deter adults from undergoing HIV Counselling and Testing, through improving the trust in healthcare system [50]. This is because HIV Counselling and Testing is an important component of the HIV prevention strategy, which involves HIV/AIDS education and recognition of one’s HIV status [51, 52]. Thus, behavioral change intervention strategies that favor PLHIV should be encouraged. Governmental policy and laws should be modified to be more accommodating to all groups, as policies that segregate a particular group may hamper on the progress of HIV interventions and programs [53]. With Nigeria a signatory to numerous of human rights declarations including the Convention on Elimination of all Form of Stigma and Discrimination against Women [54]; there is the need for human rights organizations to intensify efforts in the enforcement of the anti-HIV discrimination law and ensure the conformity to these rights, with more innovative efforts towards HIV/AIDS advocacy, awareness.

### Strengths and limitations of study

One strength of the study is that the MICS questionnaire used in the survey had been validated, pretested, and modified for individual countries (including Nigeria) in collaboration with UNICEF. Another strength is that the study involved a two-stage stratified cluster sampling and the selection of the households was achieved by a systematic random sampling. Thus, the findings from this study could be generalized to the general female population of Nigeria.

There are several study limitations. The first limitation is the cross- sectional study design, which only describes the situation in the population rather than causal pathways as regards the HIV knowledge and attitude towards PLHIV. Secondly, in building the models using the variables for knowledge for the different surveys, we observed that the selected models did not fit well with the collected data based on Archer-Lemeshow statistics. This might indicate that some important covariates potentially associated with HIV knowledge and attitude were missing in our study which may include ethnicity, religion, and some other variables. Also, the cut-off level for categorizing high and low knowledge level might have been too conservative, thus this study might have underestimated the rates of high HIV knowledge level among the female Nigerian population. And the last limitation is that the reliability estimators using the Cronbach’s alpha test were low, indicating marginal reliability of estimates in the study outcomes. Further studies are needed to assess reliability of the survey in assessing HIV knowledge and attitude towards PLHIV among Nigerian women.

## Conclusions

Our study reveals a considerable increasing tendency for high HIV/AIDS knowledge and positive attitude towards PLHIV over the years. However, the southern zones fell short of knowledge level compared to the northern zones. Women’s age, wealth index, education level and residence were consistently associated with knowledge and attitude over the years. There is a need for more pragmatic HIV/AIDS-related knowledge action plan to target to cover all age groups, all geo-political zones while paying close attention to the rural areas and the less educated women. These programs and actions should be geared towards closing HIV/AIDS knowledge gap, as part of country effort in achieving the Sustainable Development Goals (SDG 3) [55] - one of which is the eradication HIV epidemic by 2030. In addition, more replicative studies of HIV/AIDS knowledge and attitude trends is crucial in monitoring of the progress of HIV interventions in the country in the coming years.

## Data Availability

The datasets analyzed in this study publicly available from the UNICEF website: https://mics.unicef.org/surveys

## Abbreviations

AIDS: Acquired Immune Deficiency Syndrome
HIV: Human Immunodeficiency Virus
MICS: Multiple Indicator Cluster Survey
MDGs: Millennium Development Goals
PLHIV: People Living with HIV
SDGs: Sustainable Development Goals
UNICEF: United Nations Children’s Emergency Fund
WHO: World Health Organization
